# High-depth RNA-seq data to investigate gene regulation mediated by phase-variable DNA methyltransferases in serogroup B *Neisseria meningitidis*

**DOI:** 10.1128/mra.00163-26

**Published:** 2026-04-29

**Authors:** Alice Ascari, Luke V. Blakeway, Michael P. Jennings, Kate L. Seib

**Affiliations:** 1 Institute for Biomedicine and Glycomics, Griffith University 5723, Gold Coast, Queensland, Australia; University of Manitoba, Winnipeg, Manitoba, Canada

**Keywords:** *Neisseria meningitidis*, DNA methylation, epigenetic regulation, Mod, RNA sequencing, Illumina

## Abstract

*Neisseria meningitidis* is a leading cause of bacterial meningitis and septicemia. Here, we report high-depth RNA-seq for *N. meningitidis* serogroup B strains MC58, B6116/77, and M0579. These strains encode phase-variable DNA methyltransferases ModA11, ModA12, and ModD1, respectively, which act as global epigenetic regulators central to meningococcal pathogenicity.

## ANNOUNCEMENT

*Neisseria meningitidis* serogroup B is a leading cause of bacterial meningitis and septicemia (1). *N. meningitidis* encodes phase-variable N^6^-adenine DNA methyltransferases (Mods) that undergo high-frequency, reversible ON-OFF switching (phase variation) (2–4). Mod ON-OFF switching generates genome-wide alterations in methylation patterns, which modulate changes in global gene expression (5). Distinct Mods possess divergent target-recognition domains (TRDs) that define their DNA methylation target, and therefore each regulate a unique repertoire of genes termed a phase-variable regulon, or phasevarion (2, 3, 6). Here, we report the release of high-depth Illumina RNA-seq data sets for three sequenced *N. meningitidis* strains, MC58 (expresses ModA11), B6116/77 (expresses ModA12), and M0579 (expresses ModD1) (3), each profiled in a Mod ON state and an isogenic *mod::kan* knockout (KO), in biological triplicate.

Strains were grown in GC broth supplemented with 30 µM deferoxamine mesylate (Desferal; Sigma-Aldrich), to mimic biological iron-deplete conditions. Cultures were grown at 37°C with aeration (200 rpm). For each strain, the wildtype and mutant demonstrated similar growth rates. Total RNA was extracted using Trizol (ThermoFisher) from mid-logarithmic phase cells, following manufacturer’s instructions. Libraries were prepared using the Illumina Stranded Total RNA Prep with Ribo-Zero Gold Plus kit. Briefly, rRNA was depleted, remaining RNA was fragmented by heat and divalent cations, and first-strand cDNA was synthesized using randomly primed SuperScript II reverse transcriptase, followed by dUTP incorporation during second-strand synthesis to preserve strandedness. Libraries were then 3′ adenylated, adapter-ligated, and enriched by PCR (13 cycles), with size/quality assessed by microfluidic electrophoresis (Agilent Bioanalyzer DNA-1000 or TapeStation D1K) and qPCR quantification prior to pooling (2 nM). Libraries were then clustered on an Illumina cBot and sequenced ona NovaSeq X Plus using TruSeq SBS v3 reagents, generating 2 × 151 bp paired-end reads. Demultiplexing produced gzipped FASTQs via Illumina DRAGEN BCL Convert (QC thresholds: ≥94% bases ≥ Q30 for all samples).

Average sequence reads for triplicate MC58 *modA11* ON and KO samples were 78,578,685 and 72,530,832; B6116/77 *modA12* ON and KO samples were 75,375,829 and 70,447,080; and M0579 *modD1* ON and KO samples were 77,057,278 and 73,053,526, respectively. Reads were aligned against reference genomes for Nm MC58 (AE002098.2), B6116/77 (CP007667.1), and M0579 (CP007668.1) using STAR (v2.3.5a) (7), producing coordinate-sorted BAM/BAI files and mapping summaries. Gene counts were analyzed with edgeR (v4.0.9) (https://bioconductor.org/packages/release/bioc/html/edgeR.html) (8) in R (v4.3.1) to compute differential gene expression values, using default TMM count normalization between samples. Counts were summarized at the gene level using the featureCounts v1.5.3 (Subread; default settings; https://subread.sourceforge.net/featureCounts.html) (9). Differential gene expression between *mod* ON and KO strains was analyzed using edgeR, and genes meeting the predefined significance thresholds (>2-fold change, count-per-million > 0.5, and false-discovery-rate < 0.05) were considered differentially expressed.

We detected >2-fold differential gene expression in strain (i) MC58, four genes were upregulated and five genes were downregulated (*modA11* ON relative to the *modA11* KO); (ii) B6116/77, two genes were upregulated and two genes were downregulated (*modA12* ON relative to the *modA12* KO); and (iii) M0579, five genes were upregulated and 11 genes were downregulated (*modD1* ON relative to the *modD1* KO). Collectively, these RNA-Seq data sets will serve as an important resource for studying epigenetic regulation in *N. meningitidis*.

**TABLE 1:** Summary of NCBI sequence read archive (SRA) Accessions for Illumina data sets under BioProject PRJNA1398541

Strain	Mod Genotype	Biological Replicates (n)	Reference assembly	SRA Accessions^*a*^	BioSample Accessions^*b*^
MC58	*modA11* ON	3	GCF_000008805.1 (ASM880v1)	SRR36698137–SRR36698139	SAMN54472394–SAMN54472396
MC58	*modA11::kan* (KO)	3	GCF_000008805.1 (ASM880v1)	SRR36698140–SRR36698142	SAMN54472397–SAMN54472399
B6116/77	*modA12* ON	3	GCF_001029815.1 (ASM102981v1)	SRR36824925–SRR36824927	SAMN54601824–SAMN54601826
B6116/77	*modA12::kan* (KO)	3	GCF_001029815.1 (ASM102981v1)	SRR36824928–SRR36824930	SAMN54601827–SAMN54601829
M0579	*modD1* ON	3	GCF_001029835.1 (ASM102983v1)	SRR36683682–SRR36683684	SAMN54453549–SAMN54453551
M0579	*modD1::kan* (KO)	3	GCF_001029835.1 (ASM102983v1)	SRR36683685–SRR36683687	SAMN54453552–SAMN54453554

^*a*^ SRA accessions are available at https://www.ncbi.nlm.nih.gov/sra?LinkName=bioproject_sra_all&from_uid=1398541.

^*b*^ Biosample accessions are available at https://www.ncbi.nlm.nih.gov/biosample?LinkName=bioproject_biosample_all&from_uid=1398541.

## Data Availability

Raw Illumina reads (FASTQ files) for *Neisseria meningitidis* serogroup B strains MC58, B6116/77, and M0579 (*mod* ON and *mod::kan*) have been deposited to the NCBI Sequence Read Archive (SRA) under BioProject PRJNA1398541. These data sets comprise raw reads, assembled transcripts were not submitted. SRA accession IDs, and the associated BioSample accessions, are summarized in Table 1.
